# White Leaf Spot Caused by *Neopseudocercosporella capsellae*: A Re-emerging Disease of Brassicaceae

**DOI:** 10.3389/fcimb.2020.588090

**Published:** 2020-10-28

**Authors:** Niroshini Gunasinghe, Martin J. Barbetti, Ming Pei You, Daniel Burrell, Stephen Neate

**Affiliations:** ^1^Centre for Crop Health, Institute for Agriculture and the Environment, Research and Innovation Division, University of Southern Queensland, Toowoomba, QLD, Australia; ^2^School of Agriculture and Environment and the UWA Institute of Agriculture, Faculty of Science, The University of Western Australia, Crawley, WA, Australia; ^3^School of Agriculture, Food and Wine, Faculty of Sciences, University of Adelaide, Urrbrae, SA, Australia

**Keywords:** *Neopseudocercosporella capsellae*, white leaf spot, Brassica, oilseed rape, cercosporin, disease resistance, disease management

## Abstract

White leaf spot can cause significant damage to many economically important Brassicaceae crops, including oilseed rape, vegetable, condiment, and fodder *Brassica* species, and recently has been identified as a re-emerging disease. The causal agent, *Neopseudocercosporella capsellae*, produces foliar, stem, and pod lesions under favorable weather conditions. *N. capsellae* secretes cercosporin, a non-host specific, photo-activated toxin, into the host tissue during the early infection process. The pathogen has an active parasitic stage on the living host and a sexual or asexual saprobic stage on the dead host. Where the sexual stage exists, ascospores initiate the new disease cycle, while in the absence of the sexual stage, conidia produced by the asexual stage initiate new disease cycles. Distribution of the pathogen is worldwide; however, epidemiology and disease severity differ between countries or continents, with it being more destructive in Subtropical, Mediterranean, or Temperate climate regions with cool and wet climates. The pathogen has a wide host range within Brassicaceae. *Brassica* germplasm show varied responses from highly susceptible to completely resistant to pathogen invasion and significant susceptibility differences are observed among major crop species. Cultural practices only provide effective disease control when the climate is not conducive. An increase in the susceptible host population and favorable weather conditions have together favored the recent rise in white leaf spot disease occurrence and spread. The lack of understanding of variation in pathogen virulence and associated resistant gene sources within brassicas critically limits the potential to develop efficient control measures.

## Introduction

White leaf spot disease caused by *Neopseudocercosporella capsellae* (Ellis & Everhart) Videira & Crous is an important disease on many Brassicaceae including oilseed, wild, vegetable, condiment, and fodder *Brassica* species (Petrie and Vanterpool, 1978; Sumner et al., 1978; Barbetti and Sivasithamparam, 1981; Cerkauskas et al., 1998; Ocamb, 2014). Recently, there has been a worldwide increase in pathogen activity and the disease is now identified as a re-emerging disease on oilseed rape and/or oriental *Brassica* vegetables, particularly in the UK (Inman, 1992; Anonymous, 2016), the USA (Ocamb, 2014, 2016) and in Australia (Van de Wouw et al., 2016; Murtza et al., 2019). While *N. capsellae* is commonly a leaf pathogen, it also produces pod and stem lesions resulting in “gray stem” disease (Petrie and Vanterpool, 1978; Inman et al., 1999). Optimum climatic conditions for white leaf spot disease development are 15–20°C (Koike et al., 2007) with frequent precipitation (Fitt et al., 1992).

The pathogen has a wide host range (Boerema and Verhoeven, 1977; Gudelj et al., 2004), infecting a variety of cultivated crucifers, including oilseed, and vegetable brassicas. Also, *N. capsellae* has been isolated from leaf lesions on “wild” or “weedy” crucifers such as wild radish (*Raphanus raphanistrum*) and wild turnip (*Brassica rapa* ssp *sylvestris*) (Marchionatto, 1947; Deighton, 1973; Morris and Crous, 1994; Francis and Warwick, 2003; Maxwell and Scott, 2008). It has also been recorded causing disease on false flax (*Camelina sativa*), a recently introduced oilseed crop in Europe (Föller and Paul, 2002), chinese cabbage, mustard type *Brassica* vegetables, and cauliflower (Lancaster, 2006). Moreover, *N. capsellae* can produce leaf spots interspersed with symptoms caused by other *Brassica* pathogens such as *N. brassicae* (Chevall.) Videira & Crous (syn. *Mycosphaerella brassicicola*), the cause of ringspot (Fitt et al., 2006), Pyrenopeziza *brassicae* Sulton & Raw, the cause of light leaf spot, and *Leptosphaeria maculans* Ces. & De Not., the cause of blackleg disease, resulting in severe disease epidemics (Thomas et al., 2019).

### The Hosts, Brassicaceae

The family Brassicaceae contains a diverse collection of economically important weeds and cultivated varieties including major sources of cooking oil, vegetables, and condiments that contribute to 12% of the world's edible vegetable oil production and 10% of the world's vegetable production (Cheng et al., 2011). *Brassica* species have broad phenotypic plasticity as demonstrated by differences between oilseed varieties, root vegetable crops such as turnip, and leafy forms such as cabbage. Among six widely cultivated vegetable and oilseed crops, three amphidiploid species: *B. napus* (AACC, 2n = 38), *B. juncea* (AABB, 2n = 36), and *B. carinata* (BBCC, 2n = 34) were formed through interspecific hybridization between three diploid *Brassica* species: *B. rapa* (AA, 2n = 20), *B. nigra* (BB, 2n = 16), and *B. oleracea* (CC, 2n = 18) (Song et al., 1988). This broad host diversity impacts on the pathogen host range, pathogen diversity, epidemiology, and disease reaction of the host-pathogen interaction.

### Economic Impacts

The impact of white leaf spot disease have become increasingly important and economically significant outbreaks have been reported on a variety of *Brassica* crops worldwide (Petrie and Vanterpool, 1978; Penaud, 1987; Inman, 1992; Ocamb, 2014). Severe losses due to white leaf spot disease can occur at the seedling stage (Ocamb, 2014) or in older plants when susceptible varieties are grown under environmental conditions favorable for disease development (Reyes, 1979; Penaud, 1987; Barbetti and Khangura, 2000). In both these situations, and particularly when the environment is conducive, white leaf spot causes significant yield losses of at least 30% in oilseed brassicas predominantly through defoliation and the development of pod lesions (Penaud, 1987; Barbetti and Khangura, 2000). Pod infections by *N. capsellae* can cause 15% yield losses in France (Penaud, 1987). A recent study in Australia demonstrated a linear decline in rapeseed yield (up to 32%) in the field with increasing white leaf spot disease incidence and severity (Murtza, You and Barbetti, unpubl). In leafy greens, the disease causes yield reductions by reducing the quality of the foliage or making them unacceptable to consumers. The disease also can cause problems in commercial operations such as mechanical harvesting (Sumner et al., 1978).

Despite white leaf spot disease being a long-standing severe disease of Brassicaceae worldwide, there are no previous reviews relating to this disease. This review evaluates research into epidemiology and disease management. Particular focus is on biological aspects to explain the basis of the recurrence of severe epidemics, the infection process, the pathogen diversity, and on the identification, deployment, and mechanisms of host resistance.

## Current Distribution

*Neopseudocercosporella capsellae* has been recorded from all subcontinents except Antarctica, over a wide range of climatic conditions from temperate to tropical, including the principal oilseed rape or mustard producing countries of the European Union, China, India, Canada, Australia, and Japan (CMI, 1986). However, the disease severity and incidence differ geographically due to variation in pathogen populations, host species, cultivars grown, different agricultural practices adopted, and prevailing local climatic conditions (West et al., 2001), in particular, temperature, humidity, and rainfall (Siebold and Von Tiedemann, 2012).

### The UK and Europe

In continental Europe, white leaf spot is widespread on oilseed rape (Perron and Souliac, 1990; Perron and Nourani, 1991; Inman, 1992; Söchting and Verreet, 2004; Krzymański, 2006), or other *Brassica* crops, including swede (*B. napobrassica*), turnip (*B. rapa* subsp. *rapa*), mustard (*B. juncea*), chinese cabbage (*B. rapa*, subspecies *pekinensis* and *chinensis*), and cabbage (*B. oleracea*) (Koike et al., 2007). The disease is most common and severe in northern Europe (Koike et al., 2007), particularly where oilseed rape is grown as a winter crop (West et al., 2001). Countries with temperate and/or mixed temperate and Mediterranean climates such as France have historically had economically damaging disease on oilseed rape (Tromas and Vincent, 1988; Perron and Nourani, 1991). Since its detection in Poland (1987), white leaf spot disease has remained a common disease on oilseed rape (Frencel et al., 1991; Starzycki et al., 2007). White leaf spot has also become increasingly important in oilseed rape crops in the UK (Anonymous, 2016). The trend toward wetter and warmer winters in parts of the UK and Europe is suggested as the cause (Inman, 1992; Rosenzweig et al., 2001).

### Canada and the USA

In Canada, white leaf spot is one of the common diseases of both oriental vegetables and oilseed rape (Petrie and Vanterpool, 1978; Petrie et al., 1985; Cerkauskas et al., 1998), and severe outbreaks have been reported (Reyes, 1979; Petrie, 1986). The major oilseed and oriental vegetable fields are in Alberta and Saskatchewan, where oilseed rape is grown during wet, mild summers that favor disease initiation and spread.

In the USA, white leaf spot is a common disease of *Brassica* crops, particularly on vegetable brassicas (Sumner et al., 1978, 1991; Kahn et al., 2007; Ocamb, 2014; Ocamb et al., 2015). For example, in the Pacific Northwest, which has a Mediterranean type climate with mild and wet winters, control of the disease is a priority (Carmody, 2017). Recently, conditions have been warmer, and with higher winter rainfall (Dalton et al., 2017), which along with the presence of a susceptible host species, could be the cause of the increase in white leaf spot. Apart from vegetable crops, current interest in brassicas as cover crops, seed crops, oilseed, and biofuel crops or in broadacre crop rotations (Claassen, 2016) has contributed to increased diversity of *Brassica* crops grown in the region (Carmody, 2017) including the highly susceptible *B. juncea* (Gunasinghe et al., 2014). In California, high white leaf spot incidence on crucifers has been associated with high winter rainfall (Campbell and Greathead, 1978). Long-term climate change predictions of warmer winters and increased precipitation, will favor greater white leaf spot disease severity and spread in the USA.

### Australia

White leaf spot has been reported from major oilseed *Brassica* growing states of Australia and is considered an important disease (Barbetti and Sivasithamparam, 1981; Murtza et al., 2018). Oilseed rape (predominantly spring *B. napus* cultivars but also *B. juncea*) is a major winter crop in the Mediterranean climatic regions through to the more temperate climatic regions (Charles et al., 2010; Hope et al., 2015). The pathogen was first found on a range of vegetable brassicas in 1956, 1984, and 1987 (Eshraghi et al., 2005), and only later on oilseed rape, *B. napus* in 1979 (Barbetti and Sivasithamparam, 1981), and on *B. juncea* in 2005 (Eshraghi et al., 2005). In more recent studies, white leaf spot was recorded as widespread and damaging on *B. napus* and *B. juncea* (Couchman and Hollaway, 2016; Van de Wouw et al., 2016; Murtza et al., 2018), as well as on oriental *Brassica* vegetables (Burt et al., 2006; Len, 2009).

There is an increasing white leaf spot disease incidence in all oilseed-growing regions (Van de Wouw et al., 2016; Murtza et al., 2018). Several changes may have led to this increase. There has been a recent increase in susceptible host populations. First, through sowing blackleg disease resistant *B. napus* varieties (Sprague et al., 2006) some of which are highly susceptible to white leaf spot disease (Eshraghi et al., 2007; Gunasinghe et al., 2014, 2016d). Secondly, through the recent wide deployment of drought tolerance but highly susceptible *B. juncea*, (Burton et al., 2003; Gunasinghe et al., 2014, 2016d, 2017a). The virulent Victorian isolates of *N. capsellae* strains may have also spread throughout Australia (Murtza et al., 2019). Finally, environmental factors may have led to conditions more favorable for white leaf spot disease.

### Asia

White leaf spot is not reported as a damaging disease on oilseed rape or vegetable brassicas in the two major canola growing countries in Asia, China, and India, although the pathogen's occurrence has been recorded (Gangopadhyay and Kapoor, 1976; CMI, 1986). In India, the pathogen was first reported on vegetable *B. rapa* in 1973 (Gangopadhyay and Kapoor, 1976). In both countries, oilseed rape is grown as a winter crop in regions with cool but dry winters (Lal et al., 2001; Wang, 2002). In China, it is mainly grown in the south across the Yangtze River valley (Yang and Zheng, 2016) and in India in the northern states such as Rajasthan, Haryana, Punjab, and Uttar Pradesh (Chauhan et al., 2011). Dry weather during the crop season, and/or possibly differences in susceptibility of the locally grown species or varieties, could contribute to the comparatively low white leaf spot incidence and severity in China and India.

In tropical Asian regions (Taiwan, Sri Lanka) or subarctic regions (Russia), *N. capsellae* has been found on *Brassica* crops or weedy brassicas (CMI, 1986). However, there are no records of significant damage to crops. In 1966, white leaf spot was recorded as a new disease in the Soviet Far East on cruciferous vegetables (Nelen, 1966). However, Jedryczka et al. (2002) reported that white leaf spot was absent during their survey of North-Western or Central Russia in 1996–2000. The prevailing harsh climatic conditions (extremely low temperatures) may not favor the pathogen survival and disease development in these regions.

## Taxonomy, Morphology, and Physiology of the Pathogen

### Taxonomy

*Neopseudocercosporella capsellae* belongs to the Cercospora complex (Cercosporoid), where differentiation within the complex is based on morphological characteristics of spores and reproductive structures (Braun, 2001). Re-evaluations of morphological characters of cercosporoid hyphomycetes based on molecular data are underway, and comprehensive revisions of some genera have been published (Crous et al., 2001; Videira et al., 2016). Within the complex, *N. capsellae* is in the class *Dothideomycetes*, the family Mycosphaerellaceae (Deighton, 1973), and the genus *Neopseudocercosporella* (Videira et al., 2016). Twenty-six synonyms are documented for this pathogen previous to the recent name change to *Neopseudocercosporella capsellae* (Videira et al., 2016). The other member of the genus, *N. brassicae* (Chevall.) Videira & Crous (syn. *Mycosphaerella brassicicola*), the cause of ringspot disease, is closely related to *N. capsellae* (Videira et al., 2016), however the two species are reproductively isolated morphospecies (Gudelj et al., 2004). Although, the two species can produce coexisting leaf spots on *Brassica* species (Fitt et al., 2006), leaf symptoms caused by each of the individual pathogens are distinguishable (Petrie and Vanterpool, 1978; Corlett, 1999). Despite this, based on recent molecular data, Videira et al. (2016) suggest that these pathogens could be synonymous, but this is currently unresolved.

The teleomorph: *Mycospharella capsellae* was first reported in 1991 (Inman et al., 1991). A full taxonomic description of the anamorph was proposed by Deighton (1973), and the teleomorph *M. capsellae* and its' sexual structures have been described by Inman et al. (1991).

### Growth and Colony Characters on Artificial Media

On artificial media, the pathogen's growth rate is noticeably slow, taking 3 weeks for a colony to reach 1–2 cm in diameter (Crossan, 1954). Slow growth is a likely reason for under-reporting of the pathogen as the disease causal agent. On a variety of culture media, it produces dark to olivaceous-gray stromatic colonies with dentate margins (Inman, 1992). Optimum growth from hyphae occurs between 20–24°C and at pH 5.5–7.0 on potato dextrose agar (PDA) (Okullo'kwany, 1987). Young colonies produce thin and hyaline hyphae becoming thick-walled, septate, brown hyphae with stroma-like or sclerotia-like structures, which give rise to conidia (Crossan, 1954). However, *N. capsellae* does not sporulate on commonly used artificial media including PDA (Miller and McWhorter, 1948; Crossan, 1954), but produces conidia when the growing colonies on V8 or distilled water agar are exposed to near-UV light around 365–370 nm (Inman et al., 1991). Petrie and Vanterpool (1978) observed and extracted a red/purple/pink pigment produced by *N. capsellae* mycelial mats. More recently, this pigment has been confirmed as the mycotoxin cercosporin (Gunasinghe et al., 2016b).

### Cercosporin—The Toxin Produced by the Pathogen

Cercosporin (4,9-dihydroxyperylene-3,10-quinon) plays a significant role in pathogenesis by *N. capsellae* (Gunasinghe et al., 2016b, 2017b). Cercosporin applied to susceptible *Brassica* cotyledons induces disease symptoms, and there is a strong positive correlation between the degree of sensitivity to cercosporin and the degree of host susceptibility to *N. capsellae* (Gunasinghe et al., 2016b). It has been proposed that the high susceptibility of the oilseed varieties of *B. juncea* (Indian mustard) from India is due to high sensitivity to cercosporin, that facilitates the early establishment of the pathogen on the host tissue (Gunasinghe et al., 2016b, 2017b). Further, across different isolates, *in vitro* cercosporin production on V8 juice agar and the level of observed virulence on *Brassica* seedlings was highly correlated (Gunasinghe et al., 2016b).

As recorded in other *Cercospora* species (Daub and Ehrenshaft, 2000), *N. capsellae* secretes cercosporin into the host tissue during the infection process (Gunasinghe et al., 2016b, 2017b), and cercosporin has been isolated from diseased lesions of 16 different hosts (Fajola, 1978) including white leaf spot lesions on oilseed rape caused by *N. capsellae* (Gunasinghe et al., 2016b). Cercosporin is a non-host specific toxin, with broad toxicity against a wide range of organisms, including bacteria, fungi, plants, and animals (Daub, 1982). Cercosporin plays a prominent role in invasion of susceptible hosts and/or assists in the interactions with other organisms on the leaf surface (Hartman et al., 1988). It belongs to a group of photosensitizing molecules that absorb light energy and convert it into a long-lived electronically excited triplet state that produces activated oxygen species (Hartman et al., 1988; Leisman and Daub, 1992; Daub and Ehrenshaft, 2000). The light-activated cercosporin damages host tissue through peroxidation of cell membranes resulting in rapid cell death and nutrient leakage. This leakage of nutrients into the intercellular spaces facilitates the pathogen's initial establishment on the plant host (Daub, 1982).

**Figure 1:**
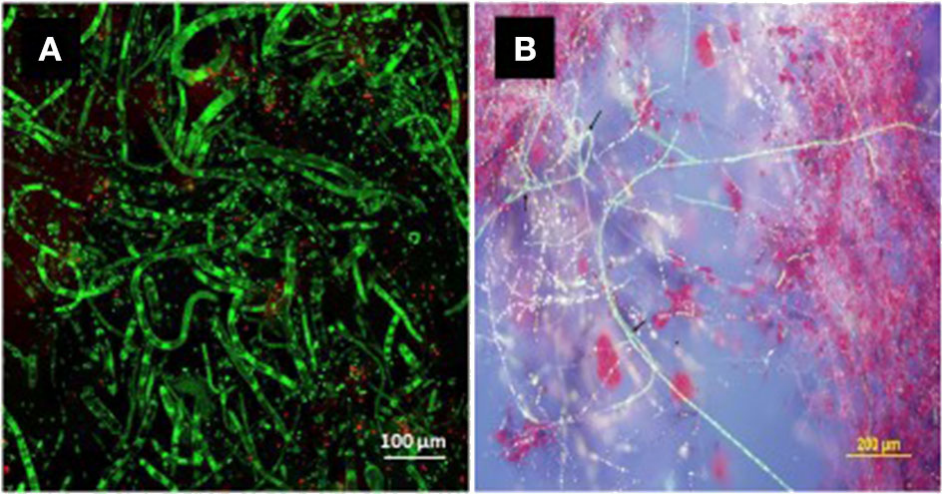
Bright green fluorescing hyphae of *Neopseudocercosporella capsellae* (isolate UWA Wlra-7) on malt extract agar detected by confocal microscopy. The green fluorescence confirms the cercosporin inside the hyphae in a chemically reduced state protecting the pathogen from its toxicity (Daub et al., 2000; Chung et al., 2002) [**(A)** cercosporin producing hyphae and, **(B)** cercosporin producing hyphae and secreted red cercosporin crystals among green hyphae].

Temperature, light conditions, and growth medium interact significantly to regulate *in vitro* cercosporin accumulation, and patterns of regulation vary among different species or even among isolates of the same species (Jenns et al., 1989). The production of cercosporin is not completely inhibited in the dark, although light is a major factor required for the process (Jenns et al., 1989; You et al., 2008). Salts, metal ions, and buffers all influence cercosporin production, but not changes in pH (You et al., 2008). The effect of temperature (Daub and Ehrenshaft, 2000) and composition of the growth medium (Fajola, 1978; Jenns et al., 1989; Gunasinghe et al., 2016a), is highly isolate-specific. While, PDA is the best medium for toxin production by other cercosporin producers (Fajola, 1978; Jenns et al., 1989), malt extract agar or V8 agar is better for *N. capsellae* (Gunasinghe et al., 2016a,b) (Figure 1). However, conclusions about whether an isolate produces cercosporin cannot be made based on cultural studies alone (Daub et al., 2000) as some isolates that do not produce cercosporin on artificial culture media, do produce it *in planta* (Upchurch et al., 1991).

## Disease Epidemiology

### Host Plant Infection and Symptoms

**Figure 2:**
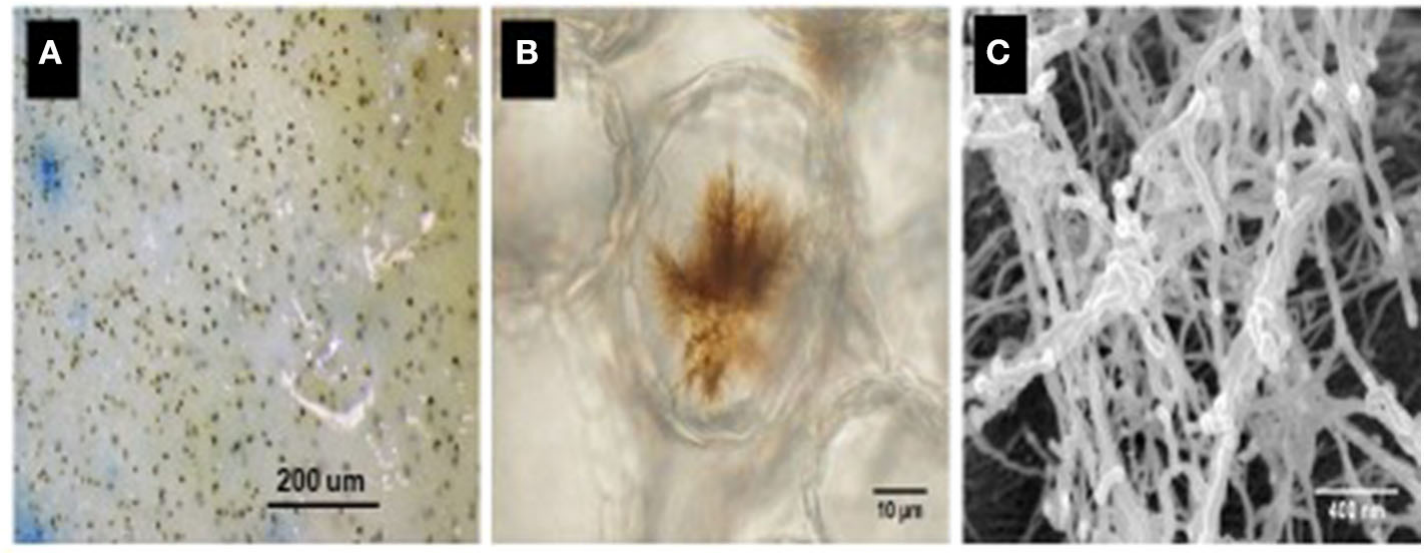
Brown infection structures produced by *Neopseudocercosporella capsellae* on a highly susceptible *Brassica juncea* cotyledon, 24 h post-inoculation as seen under a light microscope (decolorized by immersing in acetic acid: ethanol: water (2:2:1) solution at 25°C for 3–4 days) **(A,B)**. Scanning electron micrograph illustrating fine thread-like structures observed inside the host within the cortical tissue of an infected cotyledon **(C)**.

Under favorable environmental conditions, *N. capsellae* conidia (Petrie and Vanterpool, 1978; Penaud, 1987; Barbetti and Khangura, 2000) or ascospores (Inman et al., 1999) produce lesions on the leaves, while only conidia are responsible for stem and pod lesions that appear later in the crop season (Inman et al., 1991). Optimal conidial germination occurs at 20–24°C and is inhibited below 8°C or above 28°C (Crossan, 1954). Germination is usually from apical cells, or less frequently from basal cells (Morris and Crous, 1994). The conidia can produce multiple germ tubes from each conidial cell (Petrie and Vanterpool, 1978; Gunasinghe et al., 2016c) and cleavage at a septum of a conidium can produce multiple conidia, each producing a germ tube to infect the host (Gunasinghe et al., 2016c). Hyphae of germinating spores invade the host tissue through natural openings such as stomata (Crossan, 1954; Gunasinghe et al., 2016c). Temperatures of 18–19°C and high humidity (100%) with at least 8 h of continuous leaf wetness are ideal for white leaf spot infections (Inman, 1992). During the early infection stage on highly susceptible host species/cultivars, *N. capsellae* produces unique brown structures that contain cercosporin. These brown structures form highly branched networks on the cotyledon surface and/or internally within the leaf cortical tissue (Gunasinghe et al., 2017b) (Figure 2). After successful infection, the appearance of disease symptoms on oilseed rape occurs within 6–8 days at the optimum temperature range of 15–20°C and the pathogen takes longer to produce lesions below optimum temperatures (Perron and Nourani, 1991; Inman et al., 1997).

**Figure 3:**
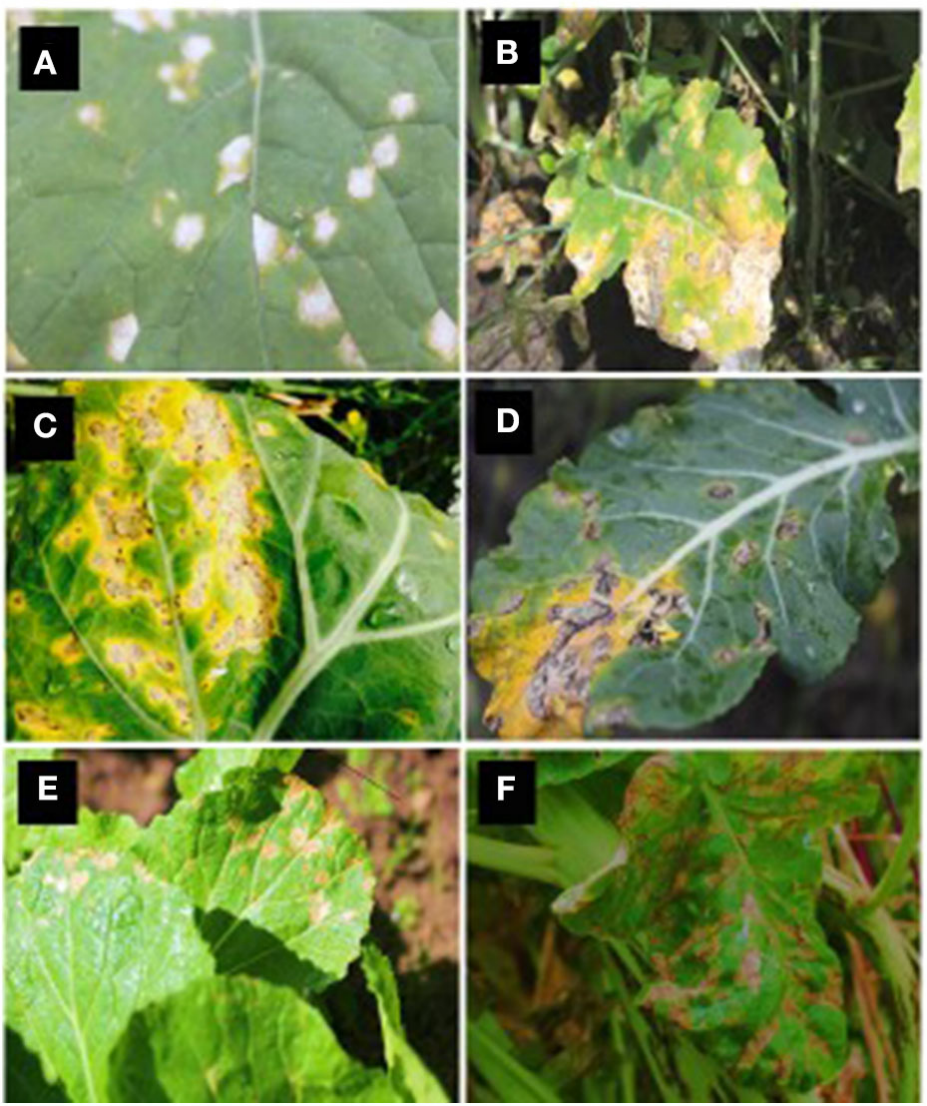
White leaf spot symptoms caused by *Neopseudocercosporella capsellae* on different hosts. Leaf lesions on oilseed rape: *Brassica napus* **(A)** and *B. juncea* **(B)**, vegetable brassicas: *B. oleracea var. capitata* (cabbage - **C**), *B. oleracea var. italica* (broccoli - **D**), *B. campestris* var. *chinensis* (Chinese cabbage - **E**), and *Raphanus sativus* (radish - **F**).

White leaf spot lesions first appear on older lower leaves, and severe infections may induce leaf fall (Barbetti and Khangura, 2000). The symptoms on brassicas can vary depending upon the degree of leaf surface waxiness or the host species (Crossan, 1954). In particular, on oilseed rape, *N. capsellae* initiates infections on leaves observable as numerous brown, elongated spots that later become white or pale beige (Figure 3A). These leaf lesions can be up to 1 cm in diameter and subsequently coalesce to form large irregular shaped lesions (Figure 3B). The mature lesions, usually surrounded by a chestnut-brown margin with a distinct delimitation between healthy and diseased tissues, are visible on upper and lower leaf surfaces. The pathogen produces evenly distributed tufts of conidiophores on upper and lower surfaces (amphigenous) of the diseased leaf (Deighton, 1973).

Related *Brassica* pathogens can also develop similar symptoms on leaves. Ringspot disease symptoms caused by *N. brassicae* could be misleading particularly when developed under dry conditions in the absence of spermogonia and/or perithecia in typical zonate arrangement (Petrie and Vanterpool, 1978; Corlett, 1999). On oilseed rape, the early development stage of blackleg caused by *L. maculans* can resemble white leaf spot, as leaf lesions are first visible as brown discolored areas. However, white leaf spot lesions are easily distinguished from mature blackleg lesions, which have small black pycnidia within the lesions and exhibit shot-holes when they age (Brun et al., 1997). Light leaf spot disease of brassicas caused by *P. brassicae* causes irregular lesions with white spore pustules (Claassen, 2016) that can be misidentified as white leaf spot disease. However, older light leaf spot lesions are much larger than *N. capsellae* lesions and have a bleached and papery center, and the infection of the leaf can cause leaf distortion (McCartney and Lacey, 1990).

The leaf spot symptoms in *Brassica* species with waxy leaves (cabbage) or turnip, mustard, and chinese cabbage varieties, differ from each other and may not resemble the white leaf spot lesions described on other hosts (Crossan, 1954) (Figures 3C–F). For example, on cabbage, initial dark gray or black dendritic lesions may resemble downy mildew symptoms caused by *Hyaloperonospora parasitica* (Pers.), but mature white leaf spot lesions become more or less rectangular or rounded with well-defined margins with ashy black center (Miller and McWhorter, 1948). In contrast, typically round, semi-transparent larger lesions with brownish-gray centers with well-defined brown or tan margins are typical symptoms on turnip and wild mustard (Miller and McWhorter, 1948).

**Figure 4:**
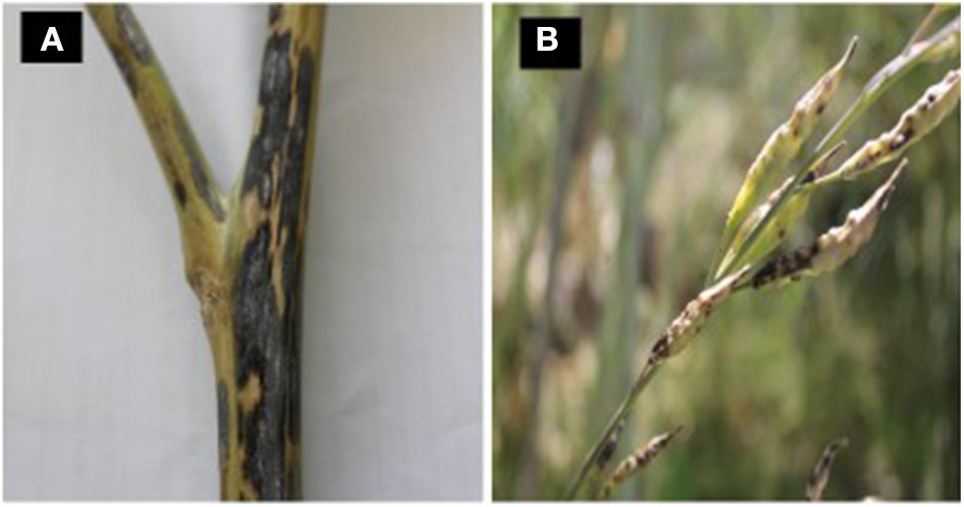
Stem and pod lesions caused by *Neopseudocercosporella capsellae* on *Brassica nigra* **(A)** and *B. juncea* **(B)**.

The pathogen can colonize stems and pods as the crop matures. The infection of stems or pods initially results in gray to black lesions (Figure 4). Geographic areas in which the sexual stage occurs, numerous tiny dark specks appear within the gray-speckled areas due to the formation of the sexual stage (Petrie and Vanterpool, 1978). These stem lesions are somewhat superficial and do not damage the pith (Inman, 1992). Pod lesions can be mistaken for *Alternaria* spp. lesions. However, *N. capsellae* lesions have dark reticulation within the brown pod-spot and a less well-defined margin (Lane and Gladders, 2008) (Figure 4). These pod lesions can expand rapidly to cover large areas with depressed centers (Carmody, 2017). By harvest, plants can become completely discolo‘red with the entire field of plants appearing purple or gray (Anonymous, 2020).

### Disease Cycle and Survival Mechanisms

During a typical disease cycle, *N. capsellae* maintains the infection chain by surviving on crop debris as a saprobe (Penaud, 1987; Inman, 1992; Barbetti and Khangura, 2000). Less frequently, the pathogen moves from a crop to a susceptible weedy host (green bridge) (Petrie and Vanterpool, 1978) such as *R. raphanistrum* (wild radish) (Gunasinghe et al., 2016a) and wild mustard (*Sinapsis arvensis*) (Miller and McWhorter, 1948) and back to a crop.

The saprobic stage of *N. capsellae* could be either resting structures produced by the asexual stage (Petrie and Vanterpool, 1978; Penaud, 1987; Barbetti and Khangura, 2000) or the sexual stage (Inman et al., 1991). Simultaneous occurrence of stromatic hyphae that produce conidia (Barbetti and Sivasithamparam, 1981; Penaud, 1987) and the sexual stage that produces ascospores (Inman, 1992) has not been recorded. Nevertheless, one or the other is responsible for the pathogen survival in different localities. The pathogen, *N. capsellae* is, therefore, capable of completing its disease cycle with (Inman, 1992), or without the sexual stage (Penaud, 1987; Barbetti and Khangura, 2000).

When the sexual stage is present, separate roles for ascospores and conidia are proposed, and there is no concurrent occurrence of both spore types (Inman et al., 1999). The sexual stage is monocyclic and is only initiated at the end of the crop season on hard crop residues such as stems, racemes, or pods, but not on leaf residues or green tissue, with ascospores released early in the next crop season (Inman et al., 1991). These ascospores require both wetness and light for induction. They are usually released in response to dew or rain, and occur within a diurnal cycle that peaks between 05.00–19.00 h. These air-borne ascospores are responsible for the first appearance of the disease in newly sown crops in autumn (Inman, 1992).

Secondary disease spread is by asexual splash-dispersed (Fitt et al., 1992) conidiospores produced on mature leaf lesions. Rain splash can only deliver conidia up to 10–20 cm vertically (Walklate et al., 1989), and therefore, the vertical progress of the disease can be halted by several weeks of dry weather during the stem elongation period (Inman et al., 1993). The asexual stage is polycyclic, the pathogen completes several life cycles in one cropping season (Inman, 1992). Development of the sexual stage is the primary survival mechanism in the UK, and failure to produce adequate stem or pod lesions would be a limiting factor for the pathogen's survival and subsequent disease carryover (Inman et al., 1993).

**Figure 5:**
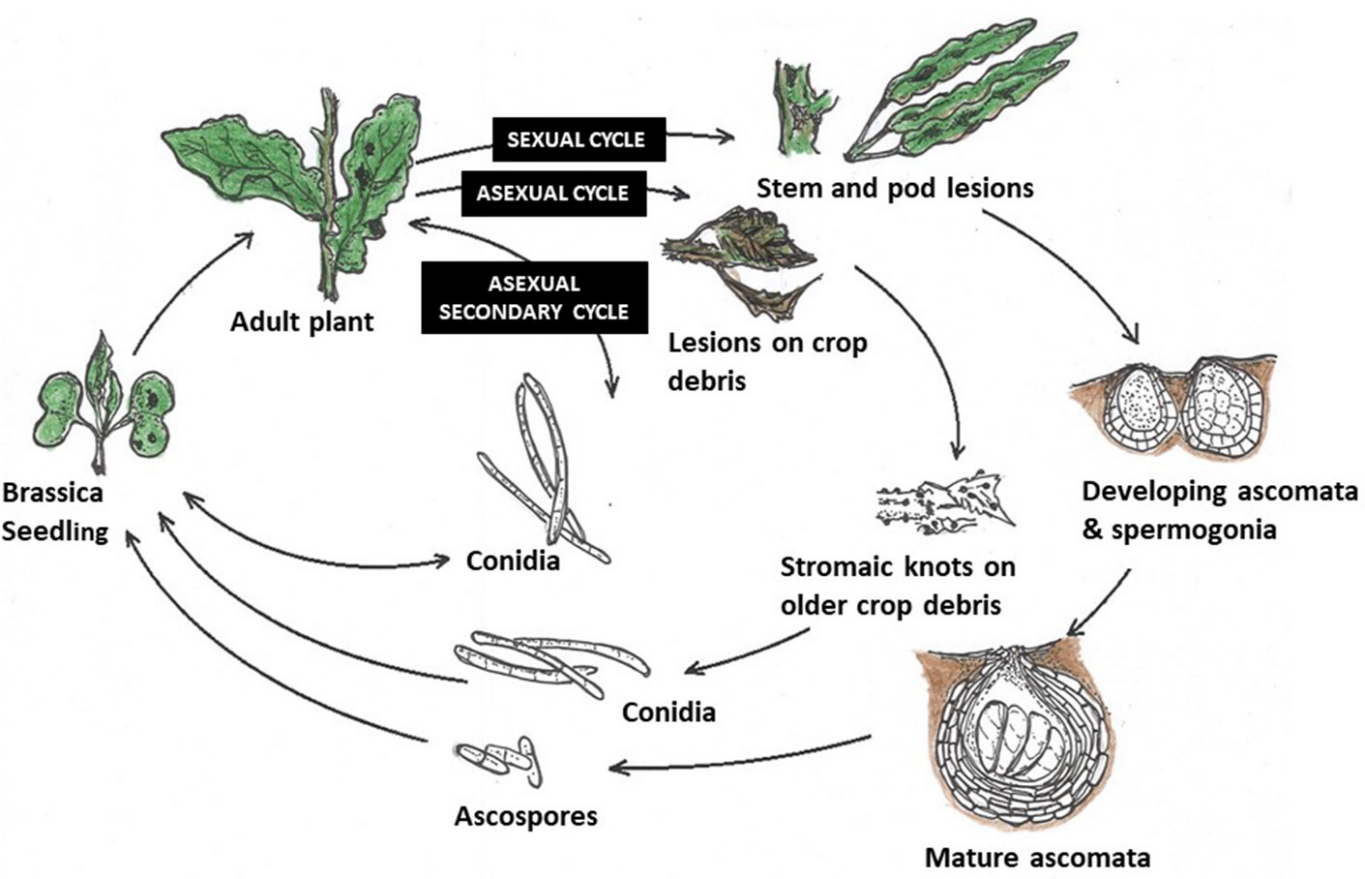
Life cycles of *Neopseudocercosporella capsellae*: (i) Sexual cycle—as found in the UK on oilseed rape (Inman et al., 1999)—Initial infection is by ascospores. The first symptoms on leaves are by contamination from air-borne ascospores produced by mature ascomata. Mature lesions on leaves then produce conidia, which are splash-dispersed and responsible for secondary infections within the crop. Sexual structures on stem and pod lesions develop and release ascospores in the following crop season. (ii) Asexual cycle—proposed life cycle for *N. capsellae*, without the sexual stage (initial infection is from conidia) as happens in Australia (Barbetti and Khangura, 2000), Canada (Petrie and Vanterpool, 1978) and France (Penaud, 1987). In the absence of any sexual stage, carryover to initiate new epidemics is likely from splash-dispersed conidia produced by infested crop residues. (iii) Secondary cycle - secondary disease spread within and between crops is reliant upon splash-dispersed conidia produced on mature leaf lesions initiated by either ascospores or conidia.

Elsewhere, in the absence of the sexual stage, *N. capsellae* survives the intercrop season through asexual resting structures commonly found on crop debris (Petrie and Vanterpool, 1978; Penaud, 1987; Barbetti and Khangura, 2000), including leaf remains (Crossan, 1954). Dark brown, vacuolated hyphae with larger cells bearing large nuclei (Inman, 1992) present in older parts of the stem or leaf lesions are responsible for producing these resting structures known as stromatic mats (Petrie and Vanterpool, 1978) or stromatic knots (Reyes, 1979). These stromatic mats are capable of producing conidial inoculum under favorable conditions to infect autumn/winter sown crops in Australia and France (Penaud, 1987; Barbetti and Khangura, 2000) or summer-sown crops in Canada (Petrie and Vanterpool, 1978) (Figure 5). Crossan (1954) demonstrated that *N. capsellae* persists as thick, dense mycelium under the epidermis, for at least 9 months on turnip leaf residues during the hot, humid summers, and cool, wet winters in North Carolina, USA. The impact of different environmental conditions on the survival rate of these stromatic hyphae to produce conidial inoculum is unknown.

### Teleomorph (Sexual) Stage Occurrence

*Mycosphaerella capsellae*, the sexual stage of *N. capsellae* naturally occurs in the UK (Inman, 1992). However, whether *N. capsellae* is heterothallic or homothallic is unknown. All attempts to produce ascospores with different isolate combinations from the UK only showed development up to the spermatial stage and did not form ascospores under laboratory conditions (Inman et al., 1991). The sexual stage has not been found in other European countries. This occurs with some other *Brassica* foliar pathogens. For example, the production of the sexual stage of *P. brassicae* (light leaf spot) is frequent in the UK, but absent in Germany (Siebold and Von Tiedemann, 2012) or Poland (Karolewski et al., 2004). Similarly, the sexual stage of *Alternaria* spp. (Alternaria blight) is seldom found in Germany, but is frequent in the UK (Siebold and Von Tiedemann, 2012). The widespread climatic variations in oilseed growing areas across these regions may cause these differences (Karolewski et al., 2004).

The only report of the sexual stage of *N. capsellae* outside of the UK is in Canada, where the spermatial stage on stems of *Capsella bursa-pastoris* was identified but did not progress to the next stage to complete the sexual cycle (Petrie and Vanterpool, 1978). Inman et al. (1991) reported that the stromatic knots in older lesions are the primodia for ascomata or spermogonia, which is the initial stage of the sexual cycle. These primordia can possibly switch from the asexual stage to the sexual stage under particular conditions. However, the conditions required for the progression of the spermatial stage into spermogonia or ascomata are unknown (Inman et al., 1991). Unlike in Europe, UK, and Australia, oilseed rape in Canada is grown in summer (West et al., 2001) and the sexual stage should, therefore, be able to resist harsh winter weather conditions such as subzero temperatures and snow cover, to be able to release ascospores after the sexual stage. However, climatic differences across the seasons in Canada may not meet the requirements of the different stages of the *N. capsellae* sexual cycle.

It is still possible that there is a cryptic sexual stage taking place in Australia or continental Europe as there have been no systematic surveys to determine the existence of the sexual stage in these regions. Inadequate understanding of the genetic basis of initiation of sexual reproduction, and environmental conditions required to develop sexual structures, limit prediction of the existence of the sexual stage. Therefore, the presence of the sexual stage in some continents/countries, including Australia, remains uncertain.

### Pathogen Dispersal and Disease Spread

Current literature suggests, where the sexual stage does not occur, that the less efficient conidial dispersal mechanism could limit disease initiation, development, and spread, as both, initial infection and secondary disease spread depend on short distance splash-dispersed conidia (Petrie and Vanterpool, 1978; Penaud, 1987; Barbetti and Khangura, 2000). However, *N. capsellae* conidia have been readily collected in spore traps positioned 1 m or more above infested residues and even in adjacent fields, suggesting that some air-borne conidial dispersal also occurs over greater distances than predicted from rain-splash alone (MJ Barbetti unpubl.).

Primary pathogen dispersal media for *N. capsellae* are rain (Fitt et al., 1992) and wind (Inman et al., 1999). In the UK, the introduction of the disease into adjacent locations is likely to be via air-borne ascospores that can travel longer distances (Inman et al., 1999). In areas where only conidia are produced, pathogen introduction over short distances would likely be through rain splash (Fitt et al., 1992), and over moderate distances by water, soil, animals (Crossan, 1954), and, as suggested above, possible short distance air dispersal. Long-distance dispersal is likely to be by infected seed, infected crop residues, machinery, and people (Crossan, 1954; West, 2014). However, two independent studies by Petrie and Vanterpool (1978) and Carmody (2017) found no evidence for transmission of disease through infected seeds.

## Disease Resistance Mechanisms

**Figure 6:**
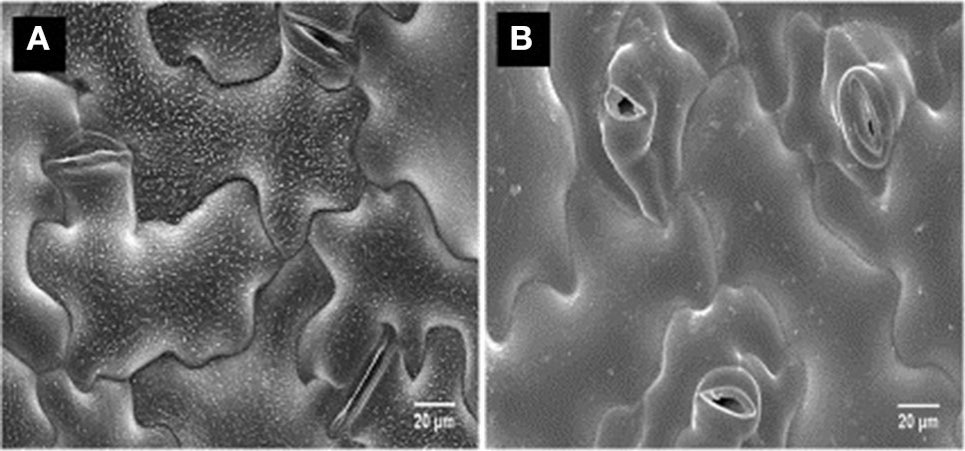
CryoSEM of a susceptible vs. a resistant genotype of *Brassica carinata*, after a challenge inoculation of the cotyledons with *Neopseudocercosporella capsellae*: highly resistant *B. carinata* genotype ATC94129P **(A)**, highly susceptible *B. carinata* genotype UWA#012 **(B)**.

Limited information is available on disease resistance mechanisms associated with *N. capsellae*. Gunasinghe et al. (2016c) named at least two possible resistance mechanisms against pathogen invasion in highly resistant *B. carinata* genotype ATC94129P. The first is where conidia on the cotyledon surface rapidly disintegrated leading to poor conidial germination, and it was hypothesized that this effect was due to physical or chemical factors related to epicuticular wax crystalloids (Figure 6A). The second is where rapid stomatal closure occurs upon pathogen recognition (Figures 6A,B) and low stomatal density limits the number of entry points for the pathogen, as *N. capsellae* does not produce appressoria or special infection structures that enable direct penetration of the host plant epidermis (Crossan, 1954; Gunasinghe et al., 2016c). In *B. juncea* and *B. napus*, impeded germination, growth, and penetration in less resistant varieties resulted in reduced infection rates and consequently less disease development. Apart from low stomatal density, the degree of leaf wettability is another non-biochemical character responsible for resistance in oilseed rape against *N. capsellae* (Inman, 1992).

Although some pre-invasive host resistance mechanisms are known for *N. capsellae*, a detailed histopathological study is needed to understand possible post-invasion resistance mechanisms. For instance, hypersensitive reactions (Li et al., 2003) and lignin formation (Sharma et al., 2008) limiting the growth of the pathogen inside the host tissue (Li et al., 2008), have been reported against other *Brassica* foliar pathogens. Further, the specific details of the biochemical and molecular events occurring on host plant tissues during disease progression in *N. capsellae*—*Brassica* spp. pathosystem remains largely unknown with no study describing the biochemical changes in the host that accompany challenges by this pathogen. Systematic studies to understand biochemical mechanisms and molecular pathways responsible for *N. capsellae* resistance in highly resistant species such as *B. carinata* will offer new avenues for enhancing resistance in susceptible species.

## Pathogenic Specificity Within *N. capsellae*

In a gene-for-gene pathosystem, pathogens have specific races/pathotypes that are compatible with resistance genes in the host. In these cases, knowledge of race structure is critical in developing and deploying host resistance. The pathogens most likely to overcome genetic resistance are those with greater evolutionary potential, have a mixed reproduction system, a high potential for genotype flow, large effective population sizes, and high mutation rates (McDonald and Linde, 2002).

Little is known about the existence of races in *N. capsellae* worldwide. In Australia, it is not known if the pathogen is endemic or exotic. Recent investigations of Western Australian isolates (Gunasinghe et al., 2016a) and Australia wide isolates (Murtza et al., 2019) revealed wide-ranging genetic and/or pathogenic variation present in *N. capsellae*, more than was expected in the absence of any known sexual stage. Genetic analysis of Western Australian pathogen isolates using its rDNA sequencing and phylogenetic analysis identified two genetically distinct populations: isolates recovered from *Brassica* crops and the isolates recovered from the weedy species *R. raphanistrum* (Gunasinghe et al., 2016a). A comprehensive study in 2015–2016 (Murtza et al., 2019) revealed significant variation among isolates from Western Australia, Victoria, and New South Wales based on its rDNA sequencing. There were also differences between isolates collected in 2015/2016 and those collected in 2005 (Murtza et al., 2019). The Australian isolates clustered separately from the North American, European, and Asian isolates (Gunasinghe et al., 2016a; Murtza et al., 2019).

Current data support the hypothesis that *N. capsellae* populations are rapidly evolving, both in genetic and virulence diversity and/or migration is occurring. These changes are agriculturally important as previously resistant cultivars become susceptible in Australia (Murtza et al., 2019). Mutation, migration, and recombination through nuclear or cytoplasmic exchange and parasexuality are likely to cause novel variations in asexually reproducing pathogen populations (Burdon and Silk, 1997). Of these, spontaneous mutation has shown strong effects on the diversity of clonal populations. Mutation was responsible for all new genetic variation within clonal lineages of *Phytophthora infestans* (Goodwin et al., 1995). As *N. capsellae* is likely to be exotic to Australia, diversity of the founder population and/or possible multiple introductions in the past could have affected the diversity of the current pathogen population. It is also possible that a cryptic sexual stage could be occurring in Australia, and a systematic search of crop and non-crop hosts should be conducted to test this hypothesis. Currently, the only known country to have mixed sexual and asexual reproduction systems is the UK (Inman, 1992), and a comprehensive comparison of the UK and isolates from other regions would be useful. However, in two studies based on its rDNA sequencing, Australian isolates were distinct from isolates in other countries (Gunasinghe et al., 2016a; Murtza et al., 2019). In another study using its rDNA sequencing, Oregon isolates showed 99.0% similarity with a single UK isolate (Carmody, 2017), which may be due to a single introduction into the USA, assuming the UK isolate was representative of its population. However, there are no studies on *N. capsellae* to understand or define the genetic variability between countries or continents, nor to determine the center of the origin or long-distance pathogen dispersal patterns.

The study by Gunasinghe et al. (2016a) demonstrated significant differences in virulence of *N. capsellae* isolates within Western Australia, with substantial variation depending upon the particular isolate × host combination. Percentage disease indices caused by 52 different isolates were within the range of 49.7–37.9 for highly susceptible *B. juncea* (cv. Rohini), and 22.7–4.5 with moderately resistant *B. napus* (cv. Trilogy). Murtza et al. (2019) confirmed that variation in virulence occurs widely among *N. capsellae* isolates across Australia. A study in Oregon, USA reported significant variation in virulence among nine white leaf spot isolates (Carmody, 2017). More extensive screening studies would show if pathotype/race-specific resistance(s) were present. If they are present, the development of a standard host differential set would enable characterization of existing pathotypes/races of *N. capsellae*. Such studies are critical for breeding of cultivars with effective and durable resistance.

## Disease Management

While host resistance is generally the preferred and most cost-effective option for disease management, cultural controls, such as residue removal, crop rotation, good hygiene practices, and fungicides can all be utilized to manage white leaf spot disease, providing conditions are not conducive for severe disease development.

Recommended cultural practices to prevent white leaf spot epidemics are typically aimed at reducing initial levels of inoculum or the spread of the established pathogen. These cultural disease control methods are preventive and indirect in action against the pathogen, and therefore, awareness of the biology of the pathogen facilitates the disease management practices by identifying the most vulnerable stage/s of the pathogen to attack (Ogle and Dale, 1997). Although, research to support the effect of cultural methods on white leaf spot disease are absent, destroying crop residues, and crop rotation are recommended to reduce the inoculum levels of necrotrophic pathogens with similar biology and survival mechanisms on crop residues (Bokor et al., 1975; Ogle and Dale, 1997).

Crop rotation, the most successful and widespread disease control practice, does provide pathogen control. This practice has been highly effective when the pathogen's survival period on the susceptible host or host residues is shorter than the rotation intervals between susceptible crops (Ogle and Dale, 1997). As *N. capsellae* has a survival period on crop residue of <1 year (Crossan, 1954), a 2–3-year crop rotation should be effective in controlling white leaf spot (Duff et al., 2006). However, other important oilseed rape pathogens such as *L. maculans* can survive longer, so 4 years between canola crops is recommended for Australia, Canada, and Europe (Bokor et al., 1975; West et al., 2001; Kutcher et al., 2013), and a minimum of 4–5-year rotations without crucifers in the USA (Carmody, 2017). Hence, there is a need to define *N. capsellae* survival periods across different continents and climates.

Stubble management is highly recommended for *N. capsellae* (Koike et al., 2007). Deep plowing of stubble reduces pathogen inoculum levels in the UK (West et al., 2001) and the USA (Ocamb, 2016). Intensive flailing and burial of residues are among the recent recommendations in Oregon to control foliar diseases, including white leaf spot (Ocamb, 2016). In Australia, stubble burning is also beneficial in controlling oilseed rape diseases, including white leaf spot (Bokor et al., 1975; Duff et al., 2006), however, the risk of erosion and reduced organic matter inputs are increased. Further, maintaining proper hygiene by removing alternative hosts (volunteer host plants, weeds, or wild relatives) would reduce inoculum carryover (Duff et al., 2006). Other strategies, such as manipulation of sowing and harvesting practices, could be used to protect the most susceptible growth stage of the crop from pathogen infection (Ogle and Dale, 1997). For instance, early or delayed sowing so that plants develop during a dry period will reduce the seedling infection rate by *N. capsellae*.

Greater spacing within and between rows reduces the potential of white leaf spot disease spread within-crop by modifying the microenvironment to reduce disease development and spread (Carmody, 2017). Overhead irrigation facilitates the spread of foliar pathogens by inoculum splash and increasing periods of leaf wetness, and high humidity, that all favor infections (Ogle and Dale, 1997). As *N. capsellae* depends on short distance splash-dispersed conidia for secondary disease spread, minimizing overhead irrigation is important. Avoiding susceptible varieties is a useful disease control strategy. Widespread planting of highly susceptible genotypes increases losses in the current season and provides high levels of inoculum for the following seasons. Host susceptibility to *N. capsellae* is increased under nitrogen deficiency, while a balanced supply of nitrogen decreases the disease potential (Duff et al., 2006).

Generally, fungicide treatment is not recommended to control white leaf spot disease in the absence of any other disease, as fungicides may not be cost-effective (Inman, 1992). However, in severe epidemics or when multiple diseases co-occur such as blackleg together with white leaf spot, application of fungicides can be beneficial and economic. If conducive weather continues after initial infections, fungicide applications are recommended every 2–3 weeks to prevent disease spread (Ocamb, 2016). If white leaf spot disease was present in the previous season, foliar fungicide application before the main rains early in the season can reduce or even prevent the disease, and monthly fungicide applications throughout the season will reduce the carryover of the pathogen in infected residues (Ocamb, 2016). In general, fungicides effective on other foliar pathogens will also reduce *N. capsellae* (Ocamb, 2016). Foliar fungicides that effective on white leaf spot are listed in Table 1.

**Table 1:** Fungicides reported as effective in controlling white leaf spot disease on *Brassica* crops.

**Fungicide**	**FRAC^a^ group**	**Country**	**Crop**	**Reference**
Prochloraz	3	Germany UK	Turnip Oilseed rape	Amelung and Daebeler, 1988 Inman, 1992
		France	Oilseed rape	Penaud, 1987
Maneb	M03	USA	Turnip	Chandler, 1965; Sumner et al., 1978
Chlorothalonil	M05	USA	Turnip	Chandler, 1965; Sumner et al., 1978
Mancozeb	M03	Australia	Oilseed rape: *B. napus*	Barbetti, 1988
Iprodione	2	USA	*Brassica* and *Raphanus* seed crops (except Canola)	Ocamb, 2016
Flusilazole	+3	UK	Oilseed rape	Inman, 1992
Carbendazim	1	UK	Oilseed rape	Inman, 1992
Benomyl	1	UK France	Oilseed rape Oilseed rape	Carmody, 2017 Penaud, 1987

a *FRAC group, fungicide resistance action committee fungicide group with the potential for cross-resistance*.

With the increasing importance of *N. capsellae* (Inman, 1992; Ocamb, 2014; Ocamb et al., 2015; Murtza et al., 2018, 2019; Thomas et al., 2019), more effective and reliable control measures are needed, particularly if climate changes lead to more conducive conditions for the development of severe white leaf spot epidemics.

Breeding for disease resistance to *N. capsellae* offers the best avenue for cost-effective control of white leaf spot. However, white leaf spot has not been a priority in the past (Inman, 1992), nor is it a priority in current breeding programs, the latter due primarily because the impact and importance of white leaf spot has often been underestimated, certainly in the case of oilseed rape. Previously Brun and Tribodet (1991) optimized a method to evaluate the resistance in *B. napus*, and more recently Gunasinghe et al. (2014) developed a rapid controlled environment cotyledon screening assay to reliably identify *Brassica* germplasm resistance to *N. capsellae*. This research now allows breeders to more efficiently identify phenotype resistance in breeding programs and has prompted a resurgence of interest in developing resistant varieties.

### Variations in Susceptibility/ Resistance and Promising Resistant Sources

A wide range of variation, from highly susceptible to complete resistance, is present against *N. capsellae* within and between Brassicaceae species. Field screening in Western Australia identified significant susceptibility/resistance variation in major Brassicaceae crops and sources of resistance to a mixture of Australian isolates of *N. capsellae* (Eshraghi et al., 2007; Gunasinghe et al., 2014, 2016d, 2017a). These results clearly demonstrate the significant potential to incorporate resistance against white leaf spot disease into commercial germplasm of oilseed, forage, and vegetable cruciferous crops through effective breeding strategies.

#### Meta-Analysis to Identify Susceptibility Variations Between Species

**Figure 7:**
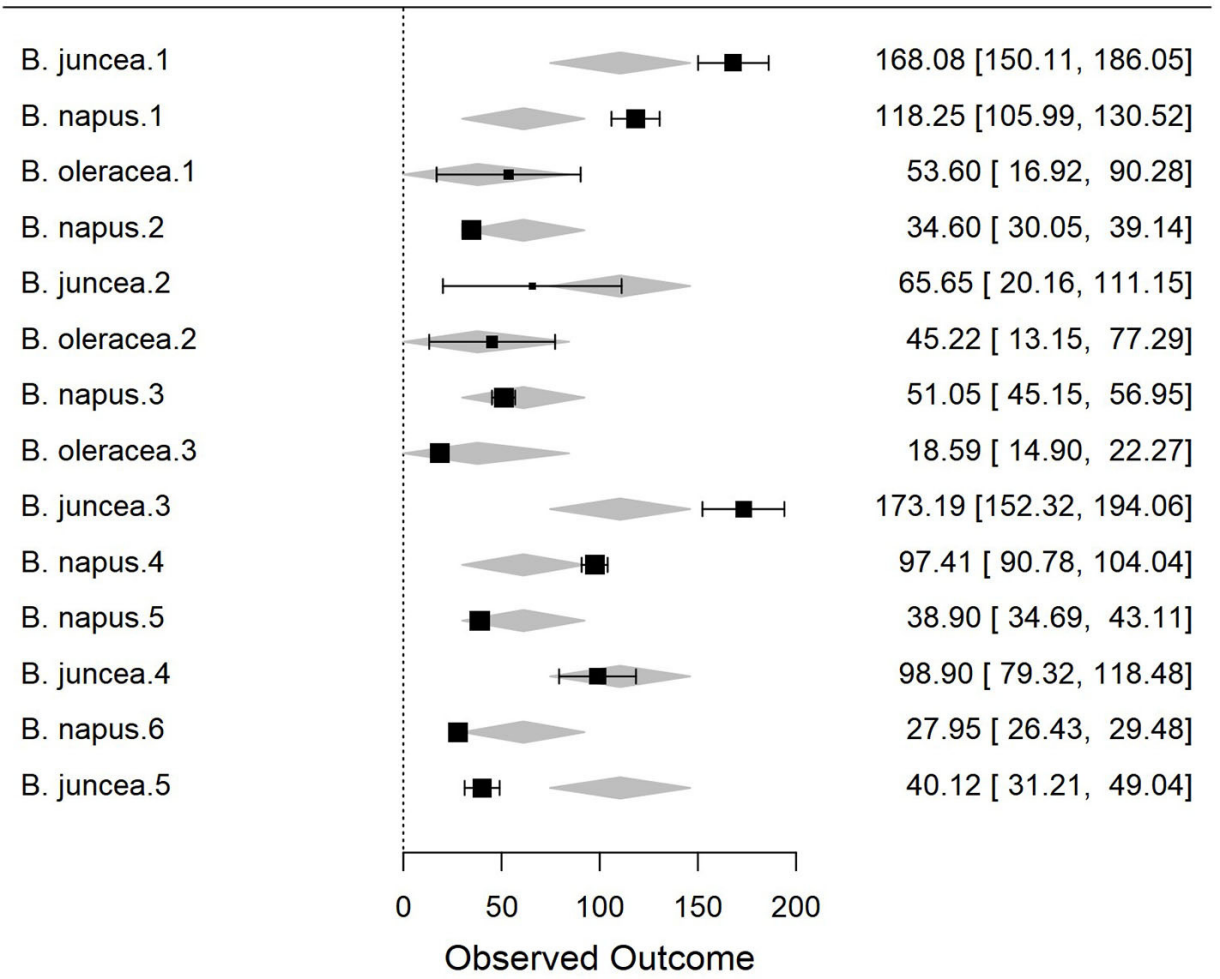
Forest plot of the meta-analysis using the random-effects model and standard REML procedure. The effect sizes of three species compared: *Brassica juncea, B. napus*, and *B. oleracea*. For individual study, outcome value or effect estimates (box) along with the confidence intervals (CI) and overall effect (diamond) are shown.

To better understand the differences in susceptibility/resistance in two common broadacre oilseed rape species (*B. juncea* and *B. napus*) and one vegetable *Brassica* species (*B. oleracea*) a meta-analysis of previously published data was conducted. Fixed and random-effects models were employed to combine data from 6 independent screening trials conducted in 2012 (Gunasinghe et al., 2014), in 2013 (Gunasinghe et al., 2016d), and in 2016 (Gunasinghe et al., 2017a) in Western Australia. Data on sample size, means, and dispersion statistics were extracted from each of the three species in each of the six studies, as listed in Table 2. A standardized measure of effect size was then calculated using this information and the random-effects model was fit using the standard REML procedure. The model outputs include estimated study effect sizes and 95% confidence intervals (CIs) for the study effects. Results indicate significant differences in resistance/susceptibility levels across the three species tested. *B. juncea* appears to exhibit greater susceptibility than the other two included species: *B. napus* and *B. oleracea* (Table 3 and Figure 7). A more comprehensive meta-analysis is required, when additional input data becomes available in the future, in order to further investigate and define this apparent effect with greater precision.

**Table 2:** Details of six field trials conducted in Western Australia, used to extract data for meta-analysis to identify susceptibility differences among three common *Brassica* species to *Neopseudocercosporella capsellae*.

**Reference**	**Number of trials**	**Method of disease assessment**
Gunasinghe et al., 2014	3	AUDPC^a^ for percentage leaves diseased
Gunasinghe et al., 2016d	2	AUDPC for percentage leaves diseased
Gunasinghe et al., 2017a	1	Percentage leaf disease index

a *AUDPC, Area under the disease progress curve*.

**Table 3:** Result of meta-analysis using random-effects model and standard REML procedure to compare disease ratings across three species, *Brassica napus, B. juncea* and *B. oleracea*, upon artificial inoculation of *Neopseudocercosporella capsellae*.

**Genus**	**Estimate**	**Standard error**	**Z**	**p**	**Lower bound**	**Upper bound**
*B. juncea* (intrcpt)	110.39	18.20	6.067	<0.001	74.72	146.06
*B. napus*	−49.28	24.16	−2.04	0.041	−96.63	−1.93
*B. oleracea*	−72.61	29.94	−2.42	0.015	−131.30	−13.93

#### Known Resistant Sources

**Figure 8:**
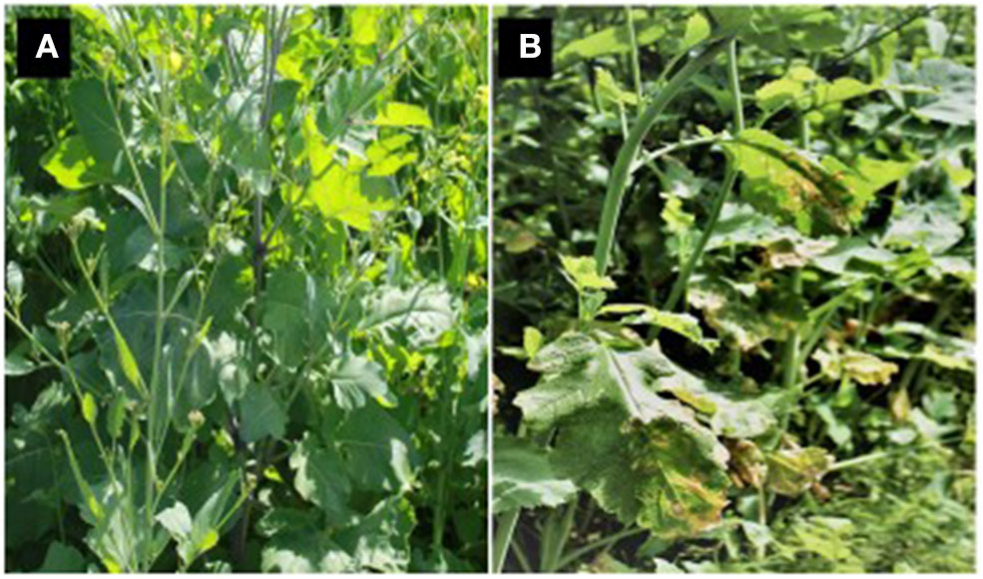
*Brassica carinata* genotype ATC94129P showing complete resistance **(A)** and highly susceptible **(B)** *B. juncea* under equal disease pressure after inoculation of a mixture of *Neopseudocercosporella capsellae* isolates.

The majority of *B. carinata* varieties screened in the above studies showed complete resistance to white leaf spot disease with at least 20 of the 32 tested cultivars/genotypes with complete resistance (Figure 8A) and with the remainder highly susceptible, suggesting a single major resistance gene involved in the resistance in that species. In contrast, the presence of genotypes with high, low, and intermediate resistance in two other species, *B. juncea* and *B. napus*, suggests resistance to these is more likely to be polygenic (Gunasinghe et al., 2014). Although Indian *B. juncea* (Figure 8B) and *B. napus* varieties were highly susceptible to white leaf spot disease, the varieties from China were markedly less susceptible under Australian field conditions (Gunasinghe et al., 2014). Therefore, the range in genetic diversity of oilseed rape germplasm can differ between India, China, and Australia, resulting in different levels of resistance for each of the three oilseed rape species depending on the country of origin.

The resistance of forage or vegetable brassicas to *N. capsellae* is variable. *B. oleracea* species display high resistance to Australian isolates (Gunasinghe et al., 2014) or to the US Pacific Northwest isolates of *N. capsellae* (Carmody, 2017). Furthermore, one or more genotypes within *B. oleracea* var. *acephala* (completely resistant), *B. oleracea* var. *capitata*, and *B. oleracea* var. *italica* (highly resistant) are excellent sources of resistance for Australia. In contrast, most Asian leafy vegetables such as *B. rapa* var. *rosularis, B. campestris* var. *chinensis, and B. rapa* are extremely susceptible to Australian isolates of *N. capsellae* (Gunasinghe et al., 2016d).

Availability of diverse sources of resistance provides a strong foundation for a breeding program to incorporate resistance into new commercial cultivars. However, it would be beneficial to have further information about resistance inheritance, identification of resistance gene/s, mapping resistance gene locations in the genome, and identification of tightly linked molecular markers (Michelmore et al., 1991; Roberts, 1992; Young, 1996). Within the Brassicaceae, the minimal incompatibility to hybridization will facilitate the use of host plant resistance (Song et al., 1988). Utilizing wild and weedy species (Uloth et al., 2013), and their introgressions into both *B. napus* (Barbetti et al., 2014; You et al., 2016), and *B. juncea* (Barbetti et al., 2014; Rana et al., 2017, 2019; Atri et al., 2019), and resistances introgressed from the B genome of *B. carinata* into *B. napus* (Barbetti et al., 2014; You et al., 2016) has been effective in developing resistance against *Sclerotinia sclerotiorum*. A similar strategy should be effective in developing brassicas with resistance against *N. capsellae*, for example, as there are both immunity and high levels of resistance found within *B. carinata* to *N. capsellae*. In addition, this species offers benefits, such as resistance to other oilseed diseases (Katiyar et al., 1986; Tonguc and Griffiths, 2004; Subramanian et al., 2005), high levels of drought tolerance (Katiyar et al., 1986; Cardone et al., 2003), and resistance to pod shattering (Salisbury and Barbetti, 2011). To prevent ongoing and future yield losses from this disease, incorporation of white leaf spot disease resistance into future varieties is urgently required. This will be even more urgent should the highly susceptible *B. juncea* be more widely adopted across canola growing regions.

## Conclusion

A lack of information exists on critical aspects of the *N. capsellae*–*Brassica* pathosystem. These deficiencies include a lack of understanding of critical components of disease epidemiology, pathogen genetics, pathogen lifecycle, and population biology. For example, comprehensive studies on population genetics and diversity would provide critical information to link the current findings of Australian pathogen populations to the global *N. capsellae* populations, to identify long-distance pathogen dispersal methods and patterns, and most importantly, fully explain the reproductive behavior of the pathogen. An appropriately designed genetic study should identify whether the population is clonal and if there is a cryptic sexual stage, define the relative degrees of sexual vs. asexual reproduction within the population by differentiating asexually reproduced clones and sexually reproduced individuals (Wang et al., 1997). Also, appropriate genetic studies will assist in defining if mating types of *N. capsellae* occur. Together with genetic studies, systematic surveys of the sexual stage, in regions where to date there are no records of the sexual stage, will resolve the current uncertainty of occurrence or non-occurrence of the sexual stage. Together, such new information will confirm and/or re-define the pathogen's disease cycle in the absence of the sexual stage and provide the current missing knowledge about disease epidemics in different localities and seasons.

Currently, most of the pathogen populations, except in the UK, are clonal, suggesting that those populations have less diversity and lower evolutionary potential. However, this should not undermine the potential future effects of *N. capsellae*, as mutation and natural selection favor the rapid establishment of more virulent strains in clonal fungal pathogen populations (Burdon and Chilvers, 1982; Burdon and Silk, 1997). The need for future studies in this area is highlighted by Murtza et al. (2019), who showed that geographically diverse *N. capsellae* pathogen populations could result in differences in virulence and pathogenicity.

In the absence of the teleomorph stage in the disease cycle, disease initiation remains poorly understood. If conidia are the primary, and in many instances the only, inoculum, as proposed by the current literature, clearly there is a more substantial role for conidia in initiating and spreading the disease than has been considered to date. Currently, the larger conidia (compared with ascospores) have been assumed unlikely to be efficiently dispersed by wind (Fitt et al., 1992) and, as such, less successful than airborne ascospores for pathogen movement. Therefore, experiments are needed to definitively define the role of conidia in the disease cycle, where the sexual stage is absent. Windborne dispersal of conidia is possible, as suggested by unpublished field observations noted in this review. Studies to understand and define the spatial and diurnal movement patterns of conidia are, therefore, critical to fully understand the epidemiology of white leaf spot, particularly with environments where strong winds prevailing during the growing season, as occurs across much of Southern Australia.

Overall, existing knowledge gaps in disease epidemiology impede the potential for reliably predicting climate change effects on white leaf spot epidemics. Since precipitation is a likely critical factor controlling white leaf spot disease epidemics, a better understanding of the current and predicted future variation in local patterns of precipitation, and their consequent effects on white leaf spot epidemics, is critical to explain current and forecast future disease epidemics across different countries and continents. It remains unclear from limited available data whether forecast climate changes will create a more or a less-conducive environment for future white leaf spot epidemics in different localities and countries where brassicas are grown. Strict cultural practices, identification of more cost-effective chemical control options, and selection of more resistant varieties all offer significant opportunities for greater reduction of white leaf spot incidence, severity, and overall management. While cultural controls offer greater avoidance of infested crop residues, fungicidal control could offer immediate control options in current disease-conducive environments. However, these areas of disease management overall remain poorly understood or defined. The identification of host resistance offers the best long term and most cost-effective management of white leaf spot. While a range of effective resistance sources has been identified across different brassicas, there is little understanding of the different types of resistance mechanisms. Even more critical in terms of practical management of white leaf spot disease is the current disconnect between identified host resistances and their uptake by *Brassica* breeding programs, which needs to be addressed urgently if improved long term management is to be secured through the deployment of effective host resistance.

## Author Contributions

NG: worked with DB to perform the meta-analysis and wrote the manuscript with input from all authors. MB and MY: added extra information and involved in critical revisions. DB: performed meta-analysis, and drafted the methodology. SN: conceived the idea, helped with reading, organization and critical revisions.

## Conflict of Interest

The authors declare that the research was conducted in the absence of any commercial or financial relationships that could be construed as a potential conflict of interest.
